# Physical inactivity and barriers to physical activity among Type-2 diabetics in Kumasi, Ghana

**DOI:** 10.4314/ahs.v23i3.38

**Published:** 2023-09

**Authors:** Linda Kumah Taylor, David Adjatey Nyakotey, Alexander Kwarteng

**Affiliations:** 1 Department of Biochemistry and Biotechnology, Kwame Nkrumah University of Science and Technology, Kumasi, Ghana; 2 Tropical Infections and Non-Communicable Diseases Research Group, Kumasi Centre for Collaborative Research in Tropical Medicine, KNUST, Kumasi, Ghana

**Keywords:** Physical activity barriers, Type 2 diabetics, Ghana, physical activity

## Abstract

**Background:**

Although the benefits of physical activity (PA) in diabetes management are well documented, there insufficient data on physical activity levels and barriers to physical activity among Type-2 diabetics in Ghana. This study assessed physical activity and barriers to physical activity among Type-2 diabetics at Manhyia Hospital in Kumasi, Ghana.

**Methods:**

The study recruited 97 participants (32% men, 68% women). Physical activity was assessed using the Global physical activity questionnaire and barriers to PA were assessed using the Barriers to being active Quiz. Anthropometry and sociodemographic data were also collected.

**Results:**

Prevalence of overweight/ obesity was 63.9%. About 60% of participants were inactive. Social influence (60.8%) was the most prevalent PA barrier followed by lack of energy (59.8%) and lack of willpower (58.8%). Majority of participants (57.7%) reported at least 4 barriers to being active. There was a significant negative correlation between age and number of PA barriers (r = -0.214, p = 0.035). A significantly higher proportion of employed participants were active compared to the unemployed/ retired participants (p = 0.035).

**Conclusion:**

This population of Type-2 diabetics needs urgent lifestyle interventions to improve physical activity and weight, considering that the main physical activity barriers were personal motivation related.

## Introduction

Diabetes mellitus (DM) refers to a collection of metabolic illnesses associated with high plasma glucose concentrations as a result of defective insulin secretion and/ or insulin action 1. Type2 diabetes mellitus (T2DM) is common, accounting for about 90% of diabetes mellitus cases worldwide 2. The International Diabetes Federation (IDF) revealed that about 463 million people aged 20 to 79 years, representing 9.3% of the adult population worldwide are living with diabetes mellitus with about 19 million of this number residing in Africa 3. In a systematic review by Asamoah-Boaheng et al. 4, it was reported that 6.5% of adults in Ghana had diabetes mellitus. There is limited data on physical activity (PA) and sedentary habits among the adult Ghanaian population in general, however, Nyakotey *et al.* 5, reported that 11% of middle-aged adults in Akuse, Ghana, were physically inactive while Abubakari *et al.* 6 estimated that among West African adults, physical inactivity prevalence was about 13%. A decrease in physical activity has been attributed to increasing access to technologies that lower work- related energy expenditure, changes in transportation, recreation and entertainment- related activities as well as high occurrence of traffic and insecurity from crime 7, 8.

Overweight/obesity and physical inactivity are two of the most prominent risk factors of type-2 diabetes mellitus^2^. The World Health Organization has advocated that in order to reduce non- communicable diseases and to improve cardiorespiratory and muscular fitness, adults who are 18-64 years old should engage in a minimum of 75 to 150 minutes of vigorous intensity aerobic activity or a minimum of 150 to 300 minutes of moderate intensity aerobic activity per week 9. Previous studies have shown that physical activity exerts positive effects on glucose control 10, reduces HbA1c 11 as well as reduces post-prandial glucose, triglycerides and insulin leading to improved glycaemic reduction in persons with high body mass index 12. Physical activity is therefore a significant element in the management of diabetes that helps to improve the health status of persons living with T2DM and subsequently reduces health professionals' burden 13.

In spite of these documented benefits of physical activity to the management of T2DM, less than 35% of T2DM patients in Tamale, Ghana, were engaged in at least 30 minutes of exercise daily 14. Another study conducted at the National Diabetes Management and Research Centre at Korle-Bu Teaching Hospital, Accra revealed that 67% of T2DM patients had low physical activity level. Furthermore, only 27% of the participants exercised 4 or more times per week 15. Failure of persons living with diabetes mellitus to comply with physical activity recommendations has been attributed to some personal and environmental barriers 16 including fear of public ridicule, presence of comorbidities 17, fear of hypoglycaemia 18, busy schedules, family responsibilities 19, unavailability of professional guidance and lack of access to exercise facilities 20. Given the dearth of information on the levels of physical activity among type-2 diabetics in Ghana, this study assessed the physical activity level and barriers to physical activity among type-2 diabetics at the Manhyia District Hospital, Kumasi.

## Methods

### Study design, study site and study population

This descriptive cross-sectional study was conducted at the diabetic clinic of the Manhyia District Hospital in Kumasi, Ghana and involved all the Type-2 diabetics attending the clinic for routine management.

### Sample size determination

The minimum sample size required was determined as follows based on Cochran's formula:


$$\begin{array}{l} \text{n}={\text{t}}^{2}\;\text{x}\;\text{p}\;(1-\text{p})\\ \;\;\;\;\;\;\;\;\;\;\;{\text{m}}^{2} \end{array}$$

Where: n = sample size, t2 = confidence level at 95%, m^2^ = margin of error at 5%

p = estimated prevalence of diabetes in Ghana is 6.5% 4.

Hence:


$$\begin{array}{c} \text{n}=1.96^{2}\text{x}\;0.065\;(1-0.065)\;=\;94\\ \;\;0.05^{2} \end{array}$$

The minimum sample size required was 94

### Sampling procedure, inclusion and exclusion criteria

Participants were conveniently sampled in May, 2021. All patients who attended the diabetic clinic were approached by the researcher and two research assistants. After explaining the study, those who agreed to participate and met the inclusion criteria were recruited. Type- 2 diabetics aged at least 18 years were included in the study. Those who were excluded were pregnant Type- 2 diabetics and those who cannot walk or have complications that greatly hinder movement.

### Data collection

A structured questionnaire was used to elicit sociodemographic information and diabetes-related history from the participants. Prior to the commencement of data collection, research assistants underwent training in the administration of the questionnaire and in proper conduction of physical activity and anthropometric assessments. The interviewer-administered Global Physical Activity Questionnaire (GPAQ) version 2 21 developed by the WHO for physical activity surveillance was used to determine self- reported physical activity level of participants. The questionnaire contained 16 questions that assessed work- related physical activity, transportation to and from places- related physical activity, recreational physical activity and sedentary time.

The Barriers to Being Physically Active Quiz 22 was used to assess barriers to physical activity engagements by participants. The questionnaire was developed by the US Center for Disease Control and Prevention to assist in identifying barriers to physical activity and to increase clinicians, participants and other stakeholders' awareness leading to targeted strategies to improve compliance with physical activity recommendations. This 21-item quiz assessed the barriers to physical activity under the following seven domains: lack of time, social influence, lack of energy, lack of willpower, fear of injury, lack of skill, and lack of resources. Each domain contained 3 items and respondents rated the degree of activity interference on a 4-point scale, ranging from 0 = “very unlikely” to 3 = “very likely, giving a total score ranging from 0 to 63. A score of at least 5 for any domain meant that domain was a barrier that needed to be worked on.

The heights of the participants were measured using a stadiometer (Seca 213 mobile stadiometer, Germany) to the nearest 0.1 cm. Weight of participants were also measured using an OMRON Body Composition Analyzer to the nearest 0.1 kg with participants wearing light clothing. The same equipment computed the body mass index (BMI) for determination of nutritional status of the participants.

### Statistical Analysis

Data was entered into Microsoft Excel and analysed with the Statistical Package for the Social Sciences (SPSS) version 25. The Shapiro-Wilk test was used to assess normality of data. For categorical variables, descriptive statistics were performed and presented as frequencies and percentages. Continuous variables were analysed and expressed as means with standard deviations for normally distributed data or median with interquartile range for non-normally distributed data. Physical activity data was processed and analysed in accordance with the Global Physical Activity Questionnaire (GPAQ) version 2 analysis framework 21. Pearson's correlation and Chi-square tests were conducted to analyse associations between continuous and categorical variables respectively. P< 0.05 was considered to be statistically significant for all analyses.

### Ethical Approval

Ethical approval was granted by the Committee on Human Research Publications and Ethics of Kwame Nkrumah University of Science and Technology, School of Medical Sciences and Komfo Anokye Teaching Hospital (Ref: CHRPE/ AP/ 168/ 21). Approvals to conduct the study was also granted by The Management of the Manhyia Hospital. Participants who were recruited appended their signatures or thumbprints to the consent form.

## Results

Table 1 presents the sociodemographic and diabetes-related characteristics as well as the nutritional status of participants of the study. A total of 97 persons (68% being women) took part in the study. The mean (SD) age of participants was 58.52 (13.64) years with majority of participants (59.8%) aged below 60 years. About 64% of participants had family history of diabetes. More than 90% participants were seeing a dietician for medical nutrition therapy for diabetes. Concerning nutritional status, 31% of participants were obese.

**Table 1:** Sociodemographic characteristics, nutritional status and disease-related characteristics of participants

Characteristic	Frequency	Percentage
**Age (years)**		
Below 60	58	59.8
60+	39	40.2
Mean (standard deviation)	58.52 (13.64)	
**Gender**		
Men	31	32.0
Women	66	68.0
**Marital status**		
Single	8	8.2
Married	53	54.6
Widow(er)	26	26.8
Divorced/ separated	10	10.3
**Educational level**		
No formal education	39	40.2
Junior high	39	40.2
Senior high/ vocational	14	14.4
Tertiary	5	5.2
**Occupation**		
Unemployed	29	29.9
Self employed	49	50.5
Formal employment	10	10.3
Retired	9	9.3
**Religion**		
Christian	61	62.9
Muslim	36	37.1
**Family history of diabetes**		
Yes	62	63.9
No	35	36.1
**Diabetes duration (years)**		
Less than 2	12	12.4
2 - 5	42	43.3
> 5	43	44.3
**Dietitian consultation**		
Yes	90	92.8
No	7	7.2
**Nutritional status**		
Underweight	3	3.1
Normal	32	33.0
Overweight	32	33.0
Obesity	30	30.9

Table 2 presents the physical activity level of participants as well as barriers to being physically active. About 60% of participants were inactive. The median sitting of reclining hours per day was 7.0 (5.0- 8.0). Among the seven barriers to being physically active, social influence (60.8%) was the most prevalent while lack of resources was the least prevalent (48.5%).

**Table 2:** Physical activity level and prevalence of barriers to being physically active

Characteristic	Frequency	Percentage
**Physical activity level**		
Active	39	40.2
Inactive	58	59.8
Sitting/ reclining hours*	7.0 (5.0- 8.0)	
**Prevalence of physical activity barriers**		
Lack of time	53	54.6
Social influence	59	60.8
Lack of energy	58	59.8
Lack of willpower	57	58.8
Fear of injury	56	57.7
Lack of skill	48	49.5
Lack of resources	47	48.5
**Number of barriers**		
Less than 4	41	42.3
4+	56	57.7

* Data presented as median (interquartile range) for skewed variables

Overall, majority of participants (57.7%) reported at least 4 barriers to being physically active.

A bivariate correlation was performed between age, BMI and physical activity parameters and presented in Table 3. A significant negative correlation (p = 0.035) was found between age and number of physical activity barriers. All other associations were however not statistically significant.

**Table 3:** Correlation between age, BMI and physical activity parameters

Variable	Total PA (METminutes/week)	Sitting/reclining hours per day	Number of PA barriers
**Age**	-0.187(0.066)	0.172(0.091)	-0.214**(0.035)**
**BMI**	0.065(0.529)	-0.086(0.403)	0.104(0.313)

Data presented as r (p-value). BMI: body mass index, PA: physical activity, MET: metabolic equivalents. Correlation (bold) is significant at p- value ≤ 0.05 (2- tailed).

Table 4 presents the association between sociodemographic characteristics, anthropometry and physical activity level. A significantly higher proportion of employed participants were physically active compared to unemployed/ retired participants (p = 0.034).

**Table 4:** Association between sociodemographic characteristics and anthropometry and physical activity level

Variable	Physical activity level		*p*- value

	Inactive	Active	
**Age (years)**			
Below 60	32(55.2)	26(44.8)	0.296
60+	26(66.7)	13(33.3)	
**Gender**			
Men	15(48.4)	16(51.6)	0.127
Women	43(65.2)	23(34.8)	
**Occupation**			
Unemployed/ retired	28(73.5)	10(26.3)	**0.034**
Employed	30(50.8)	29(49.2)	
**Educational level**			
No formal education	23(59.0)	16(41.0)	0.140
Basic	27(69.2)	12(30.8)	
Senior high/ tertiary	8(42.1)	11(57.9)	
**BMI**			
Less than 25 kg/m^2^	21(60.0)	14(40.0)	1.000
At least 25 kg/m^2^	37(59.7)	25(40.3)	

Data presented as frequency (percentages). Fischer's exact test was employed unless for variables with three categories where chi- square test was employed. P- value in bold is statistically significant.

Table 5 presents the association between sociodemographic characteristics, anthropometry and barriers to being physically active. A significantly higher proportion of participants under 60 years reported lack of time as a barrier to their being physically active compared to those aged at least 60 years (p = 0.012). Again, lack of willpower was a barrier for a significantly higher proportion of under 60-year-old compared to those aged at least 60 years (p = 0.006) and for women more than men (p = 0.008). Concerning occupation, significantly higher proportion of employed participants had lack of time as a barrier to their being physically active compared to unemployed/ retired (p = 0.022). Finally, compared to those who had basic education and those who had senior high or tertiary education, a significantly higher proportion of participants with no formal education reported that lack of resources was a barrier to being physically active (p = 0.033). All other associations were not statistically significant.

**Table 5:** Association between sociodemographic characteristics and anthropometry and physical activity barriers

Variable	Barriers to physical activity						

	Lack of time	Social influence	Lack of energy	Lack of willpower	Fear of injury	Lack of skill	Lack of resources
**Age (years)**							
Below 60	38(65.5)	40(69.0)	38(65.5)	41(70.7)	31(53.4)	33(56.9)	33(56.9)
60+	15(38.5)	19(48.7)	20(51.3)	16(41.0)	25(64.1)	15(38.5)	14(35.9)
p- value	**0.012**	0.057	0.206	**0.006**	0.402	0.098	0.062
**Gender**							
Men	15(48.4)	16(51.6)	20(64.5)	12(38.7)	20(64.5)	13(41.9)	14(45.2)
Women	38(57.6)	43(65.2)	38(57.6)	45(68.2)	36(54.5)	35(53.0)	33(50.0)
p- value	0.512	0.265	0.658	**0.008**	0.386	0.385	0.670
**Occupation**							
Unemployed/ retired	15(39.5)	20(52.6)	20(52.6)	18(47.4)	25(65.8)	20(52.6)	16(42.1)
Employed	38(64.4)	39(66.1)	38(64.4)	39(66.1)	31(52.5)	28(47.5)	31(52.5)
p- value	**0.022**	0.207	0.292	0.091	0.214	0.680	0.406
**Educational level**							
No formal education	24(61.5)	28(71.8)	25(64.1)	26(66.7)	24(61.5)	23(59.0)	25(64.1)
Basic	21(53.8)	21(53.8)	23(59.0)	21(53.8)	22(56.4)	18(46.2)	16(41.0)
Senior high/ tertiary	8(42.1)	10(52.6)	10(52.6)	10(52.6)	10(52.6)	7(36.8)	6(31.6)
p- value	0.375	0.192	0.699	0.430	0.794	0.247	**0.033**
**BMI**							
Less than 25 kg/m^2^	18(51.4)	24(68.6)	21(60.0)	18(51.4)	25(71.4)	16(45.7)	17(48.6)
At least 25 kg/m^2^	35(56.5)	35(56.5)	37(59.7)	39(62.9)	31(50.0)	32(51.6)	30(48.4)
p- value	0.675	0.283	1.000	0.291	0.054	0.674	1.000

Data presented as frequency (percentages). Fischer's exact test was employed unless for variables with three categories where Chi- square tests was performed. P- value in bold is statistically significant.

## Discussion

The benefits of physical activity in the management of T2DM have been well documented 9, 11,23,24,25. However, despite these benefits, about 60% of type- 2 diabetics in this study were inactive. The World Health Organization 9 recommends that adults and older adults living with chronic conditions like T2DM perform a minimum of 150- 300 minutes of moderately intensive aerobic PA or a minimum of 75-150 minutes of vigorously intensive aerobic PA in the course of the week. This will help to halt disease progression, prevent complications and subsequently reduce health professionals' burden 13.

The study also observed that the proportion of unemployed/retired participants who were inactive was significantly higher than those who were employed (p = 0.034), suggesting that employment contributed to attaining the recommended PA levels. Kwak *et al.* 26 similarly found that in the USA, employed persons had significantly higher PA compared to unemployed persons however, the same authors found no such difference in Sweden. It has been suggested that engaging in PA increases with educational level 27,28 however, this study found no association between PA and educational level. Also, the correlation between age and physical activity was not significant.

This study revealed that barriers to physical activity were prevalent among T2DM patients. Five out of the seven domains of PA barriers recorded over 50% prevalence. The three most cited barriers in this study were social influence, lack of energy and lack of will power, the latter two being personal barriers. Personal barriers have been documented as most frequently cited barriers to PA compared to environmental barriers 16,29,30,31. With this in mind, physicians, dietitians and other health workers involved in diabetes care and management should address issues that influence personal motivation of diabetics to increase their PA engagements. Indeed, it has been reported previously among Type- 2 diabetics that medical support (i.e., physician's direct request and monitoring of patients' PA) was the second most cited motivation for attaining recommended PA levels 32. As observed in this present study, other studies have also cited social influence (i.e., lack of support from family and friends) as an important barrier to PA among diabetics 30,32,33 and also as an independent predictor of non-compliance to PA recommendations 34. One strategy to overcome the barrier of lack of social support is to join a diabetes peer group. Mphwanthe et al. 17, reported that diabetes peer support groups served as an avenue for Type- 2 diabetics to interact and share information on PA. Findings from a meta- analysis of randomized controlled trials revealed that regular contact with other diabetics significantly bettered glycaemic outcomes 35.

With respect to lack of time as barrier to PA, this study observed that Type- 2 diabetics aged less than 60 years and those who were employed were significantly affected by this barrier compared to those aged at least 60 years (p= 0.012) and the unemployed or retired (p= 0.022). Alghafri *et al.* 30 reported similar findings among Type- 2 diabetics in Oman. Lack of will power was a significant barrier for women and those aged below 60 years while lack of resources significantly affected those who had no formal education. These sociodemographic factors may be targeted for PA interventions.

The high prevalence of overweight/ obesity recorded in this study, though worrying, comes as little surprise. This is because overweight/ obesity is a major risk factor of insulin resistance and subsequent T2DM 36 and estimates show that between 50.9 to 98.6% of adults with T2DM are overweight or obese 37. Modest weight loss of 5% to 10% among overweight/ obese T2DM persons has been documented to improve blood glucose, decrease HbA1c levels, reduce cholesterol and improve blood pressure 38,39. This study also did not report any significant associations between BMI and physical activity level or physical activity barriers suggesting that participants' weight status did not influence their physical activity level or their barriers to being physically active. Similarly, Mynarski et al. 40 reported a lack of significant associations between physical activity and BMI of T2DM patients.

It is encouraging to observe that dietician consultation was very high among the study population. It has been recommended that all individuals with diabetes should be referred to a registered dietitian who is skilled and knowledgeable in giving diabetes-specific diet therapy for individualized therapy 41 as there is no one-size-fits all eating plan. Franz *et al.* 42, reported a 0.3%- 2% decrease in HbA1c for Type 2 diabetics who received diet therapy delivered by a registered dietitian.

This study concludes that physical inactivity as well as overweight/ obesity prevalence were high in this population of type- 2 diabetics. This combination of physical inactivity and overweight/ obesity is a recipe for poor diabetes management that could lead to several complications and comorbidities. Interventions including lifestyle education at diabetic clinics are needed to improve physical activity level and weight status, bearing in mind the observation that the main barriers to physical activity were related to personal motivation.

To the best of our knowledge, there is no published study that has assessed the barriers to PA among T2DM patients in Ghana. However, due to the small sample size of this study, the results cannot be generalized for the T2DM population in Kumasi or in Ghana. Secondly, estimation bias (overestimation or underestimation) could have been introduced during the assessment of physical activity. The authors consider these as limitations of the study.
